# The Obsessive–Compulsive Symptoms in Tic Disorders and the Psychometric Properties of Children’s Yale–Brown Obsessive–Compulsive Scale: An Evidence-Based Survey in a Chinese Sample

**DOI:** 10.3389/fped.2022.794188

**Published:** 2022-06-09

**Authors:** Junjuan Yan, Yi Gu, Mengyu Wang, Yonghua Cui, Ying Li

**Affiliations:** ^1^Department of Psychiatry, Beijing Children’s Hospital, Capital Medical University, National Center for Children Healthy, Beijing, China; ^2^Medical Psychology Department, The First Medical Center, Chinese People’s Liberation Army (PLA) General Hospital, Beijing, China

**Keywords:** CY-BOCS, YGTSS, obsessive–compulsive disorder, psychometric properties, tic disorders

## Abstract

**Objective:**

Patients with tic disorders (TDs) usually also have obsessive–compulsive disorder (OCD). The severity of obsessive–compulsive symptoms (OCSs) in TD is widely evaluated using the Children’s Yale–Brown Obsessive–Compulsive Scale (CY-BOCS). However, there is no survey on the severity of OCSs in patients with TD based on a Chinese sample, and the reliability and validity of the CY-BOCS in patients with TD have not been well established in China. This study examined the severity of OCSs in TDs and the psychometric properties of the CY-BOCS in Chinese pediatric patients with TD.

**Methods:**

A total of 367 patients who were diagnosed with TD [152 with Tourette syndrome (TS)] were enrolled in the Department of Psychiatry at Beijing Children’s Hospital in China. The mean age of the patients was 9.21 ± 2.06 years (range: 5–16 years). The Yale Global Tic Severity Scale (YGTSS) and CY-BOCS were used as screening tools. The psychometric properties of the CY-BOCS were assessed using Cronbach’s alpha, test-retest reliability, and construct validity.

**Results:**

The OCSs in TDs were 3.93 ± 5.15 based on the CY-BOCS in the whole sample. The older adolescent group showed higher scores than the young adolescent groups (*Z* = −3.37, *p* = 0.001). However, the young adolescent group showed a higher incidence rate of OCSs than the older adolescent group (*p* < 0.01). Men with TDs also showed a higher incidence rate of OCSs than women (*p* = 0.03). The reliability and validity analyses of the CY-BOCS showed Cronbach’s alpha and test-retest reliability values of 0.81 and 0.82, respectively. The CY-BOCS showed an acceptable level in the two-factor structure (obsession and compulsive) in patients with TD. The comparative fit index (CFI) was 0.84 for TD, 0.86 for Tourette, 0.94 for the younger adolescent group, and 0.74 for the older adolescent group.

**Conclusion:**

More OCSs were identified in the TS group and the older adolescent group with TDs. The CY-BOCS showed good psychometric properties in children and adolescents with TD, especially in younger patients with TD. OCSs might be associated with age and functional impairment of TD.

## Introduction

Tic disorder (TD) is a neuropsychiatric disorder that is characterized by recurrent tics, namely, sudden, rapid, non-rhythmic movements, or phonic productions. In our previous national survey of children’s mental disorders, the prevalence of TDs in China was 2.5% (1). The three main types of TDs are Tourette syndrome (TS), chronic motor or vocal TD, and provisional TD. TS is characterized by multiple motor tics and one or more vocal tics (not necessarily concurrently) that persist for at least 1 year (2). The worldwide prevalence of TS in children ranges from 0.3 to 0.9% (3). Tic symptoms decline significantly by adulthood (4), with a prevalence of approximately 118 patients per million adults (5).

It should be noted that when compared with other chronic motors or vocal TDs and provisional TDs, patients with TS often have more comorbidities, and these comorbidities make treatment more difficult (6). Moreover, the comorbidities of TS will cause more impairment of social functions, family functions, learning, and school problems (7, 8). Indeed, between 85.7 and 88% of patients with TS also have psychiatric comorbidities, such as attention-deficit/hyperactivity disorder (ADHD), obsessive–compulsive disorder (OCD), anxiety disorder, depressive disorders, and self-injurious behavior (SIB) (9). Although ADHD occurs in approximately 60% of individuals with TS (10), OCD seems to show a greater association with the severity of tic symptoms (11). TS has higher tic severity and comorbidity scores (compulsion, ordering, ADHD, anxiety, and SIB) than chronic motor TD (12). Notably, up to 90% of patients with TS show obsessive–compulsive symptoms (OCSs) (13). This evidence indicates the need for increased attention to OCS in patients with TDs. There were three reasons that account for this attention. First, both the OCS and TS present repetitive behaviors and often have “just right” phenomenon, which leads to difficulties in their differential diagnosis (14). Second, SIB is present in 35% of patients with TS, and the presence of SIB is associated with obsessive–compulsive behavior (15). Third, comorbid OCD of TS is associated with poorer quality of life across the self, relationship, environment, and general domains (16). Taken together, there is also a need to better understand the characteristics of OCS in patients with TS. It should be noted that the co-occurrence of TS and OCD, which appears to be common, poses a particular challenge to clinicians with respect to make treatment recommendations to families and to implement the chosen interventions (17). Therefore, we need to pay more attention to this special subtype of TD, especially the assessment of OCD in TS, which might show the benefits of the early treatments of OCD in TS.

The Children’s Yale–Brown Obsessive–Compulsive Scale (CY-BOCS) is the most widely used instrument in children and pediatric OCD studies (18). European clinical guidelines for TS and other TDs also recommend several scales (i.e., child and adult versions of the Y-BOCS, the Leyton Obsessive Inventory, and the Children’s Obsessive–Compulsive Inventory) to assess the OCS in TS (19). However, the CY-BOCS is the most recommended instrument for OCS comorbidities in patients with TDs (19). The CY-BOCS was developed from the Y-BOCS (20), with simpler language for the items when compared with that in the Y-BOCS. The CY-BOCS is considered the gold standard for assessing the severity of OCD in children and adolescents (21). However, most studies have focused on the reliability and validity of the CY-BOCS in OCD (22, 23), and a few have assessed the reliability and validity of the CY-BOCS in children and adolescents with TDs. To our knowledge, no attempt has been made to validate the Chinese version of the CY-BOCS in OCD or TDs. Further evidence is needed to determine whether the CY-BOCS has good psychometric properties in Chinese patients with TDs.

In addition, previous studies have demonstrated the importance of age in TDs (24). For example, the onset age of TD is typically 3–10 years, with the highest estimated prevalence occurring at 7–11 years. Tic severity also declines with age during adolescence, as the age of most patients with premonitory urges of tics is above 10 years old. Moreover, tic-related OCD tends to have an earlier age of the onset (24). Taken together, age might be an influential factor for TS, but the age effect of OCS in TD might need further evidence.

Therefore, this study examined the reliability and validity of the CY-BOCS in patients with TDs. We calculated the test-retest reliability, Cronbach’s alpha, and discriminant validity. Moreover, we also used confirmatory factor analysis (CFA) to test the structure of the CY-BOCS.

## Materials and Methods

### Participants

All participants enrolled were from the Department of Psychiatry at Beijing Children’s Hospital in China from 1 October 2019 to 1 January 2020. The inclusion and exclusion criteria were age 5–16 years, meeting the diagnostic criteria for TDs according to the Diagnostic and Statistical Manual of Mental Disorders, Fifth Edition (DSM-5), no central nervous system diseases, and no intellectual disability. These patients were all first onset of TDs and drug naïve, and patients diagnosed with different comorbidities were allowed.

The Ethics Committees of Beijing Children’s Hospital of Capital Medical University (Institutional Review Board number: 2018-199) approved this study, and written informed consent was obtained from the parents or guardians of the enrolled children and adolescents.

### Measures

#### Children’s Yale–Brown Obsessive–Compulsive Scale

The CY-BOCS is a semistructured scale rated by a clinician or trained interviewer to measure the severity of obsessive and compulsive behaviors during the previous week in patients with OCD aged 8–16 years. The obsessions and compulsion subtotals are derived by adding five items (time occupied, interference, distress, resistance, and degree of control, range: 0–4) related to obsessions (range: 0–20) and compulsions (range: 0–20), respectively. The total score is the sum of the obsessions and compulsion subtotals. The CY-BOCS score and the severity of OCS are positively correlated. The cutoff point of CY-BOCS is 8 (between subclinical level and clinical level). The different severity levels of CY-BOCS (both obsessions and compulsions included) are as follows: 0–7: subclinical, 8–15: mild, 16–23: moderate, 24–31: severe, and 32–40: extreme (22).

The CY-BOCS has the same structure and items as those for the Y-BOCS. The Y-BOCS has been translated into Chinese, and its psychometric properties have been tested (25). Therefore, two translators independently translated the CY-BOCS into Chinese based on the Chinese version of the Y-BOCS. Two bilingual experts were invited to check the translated version and finish the back-translated version. The translated version was then modified until it was comparable with the original English version (26).

#### Yale Global Tic Severity Scale

The Yale Global Tic Severity Scale (YGTSS) is a semi-structured scale that is rated by a clinician or trained interviewer. It was developed to assess tics observed within the previous week (27). The five dimensions included in the YGTSS are number, frequency, intensity, complexity, and interference. The total score of the YGTSS (range: 0–100) is derived by summing the tic severity ranges between 0 and 50 (motor tics range: 0–25; vocal tics range = 0–25) and the impairment rating score (range: 0–50). The YGTSS is widely used, with excellent reliability and validity for assessing children and adolescents with TDs (28).

The two raters also completed clinical assessments. The consistency coefficient between raters was 0.87. All participants were outpatients. The assessments were performed after diagnosis in the Psychiatric Department’s Psychology Assessment Room at Beijing Children’s Hospital by one of the two psychiatrists. Furthermore, a total of 30 participants were randomly selected for retesting 1 month later to verify the test-retest reliability of the CY-BOCS.

### Statistical Analysis

Statistical analyses were performed using IBM SPSS Statistics for Windows, version 25.0 (IBM Corp., Armonk, NY, United States). First, Shapiro–Wilk tests were used to assess the distribution of each item, subscale (obsession and compulsion), and total score. If the distribution of these variables was normal, *t*-tests were used to compare the differences between groups, such as gender and age. If the distribution of these variables was non-normal, the Mann–Whitney *U* test was used. Partial correlation analysis was also used to explore the age effect of OCS in TDs (controlling for the duration of illness). Second, we used Spearman’s rank correlation analyses to examine the item-total correlations of the CY-BOCS. Third, we used Cronbach’s alpha and the test-retest reliability to assess the reliability of the CY-BOCS. Fourth, the discriminant validity of the CY-BOCS was calculated using the Pearson correlation coefficients between the CY-BOCS scores and the severity scores of the YGTSS. Finally, we performed CFA of CY-BOCS using the “lavaan” package in R (version 3.5.3). Considering the age effect for OCS in TD, we also calculated the assessed CFA of CY-BOCS in different age groups. Thus, CFA was performed for the whole sample and the TS (only patients with TS), younger adolescent (age <10 years), and older adolescent (age ≥10 years) groups.

## Results

### The Clinical Characteristics of Obsessive–Compulsive Symptom in Tic Disorders

A total of 382 children and adolescents with TDs were enrolled, 15 of whom were excluded because they did not complete the YGTSS or CY-BOCS assessments. Finally, 367 patients were enrolled in the study. Among these patients, 152 patients were diagnosed with TS (41.42%) [100 (27.25%) with chronic motor or vocal TD and 115 (31.33%) with provisional TD]. The mean age of the patients was 9.21 ± 2.06 years (range: 5–16 years). The younger and older adolescent groups included 230 (62.67%) and 137 (37.33%) patients, respectively.

The Shapiro–Wilk tests used to evaluate the distributions of each item, subscales (obsession and compulsion), and total scores showed that all items, subscales (obsession and compulsion), and total scores were non-normally distributed (*p* < 0.001) (Supplementary Table 1). Therefore, we used the Mann–Whitney test to compare the differences between age groups (younger adolescents and older adolescents) and gender (males and females). We observed no difference between the men and women in obsession (*Z* = −0.17, *p* = 0.86), compulsion (*Z* = −0.48, *p* = 0.63), or total scores (*Z* = −0.39, *p* = 0.69). However, we observed significant differences between younger and older adolescents in obsession (*Z* = −3.59, *p* = 0.05), compulsion (*Z* = −1.96, *p* < 0.001), and total scores (*Z* = −3.37, *p* = 0.001).

Considering the potential influence of the duration of illness on the severity of OCS in different age groups, we used partial correlation analysis to explore the age effect of OCS in TDs while controlling for the duration of illness. The results showed a weak but significant correlation between age and the severity of obsession (*r* = 0.17, *p* = 0.001), compulsions (*r* = 0.15, *p* = 0.004), and total scores (*r* = 0.22, *p* < 0.001).

Table 1 shows the CY-BOCS, obsession subscale scores, and compulsion subscale scores for the whole sample, the TS group, and the younger and older adolescent groups. The mean [standard deviation (SD)] of total CY-BOCS for the whole sample was 3.93 (5.15). For the TS group, the mean (SD) was 4.32 (5.32). We found that 86.32% of the whole samples reported OCS. Furthermore, 36.72% of the whole sample had at least a mild level of OCS (cutoff score 8), 15.21% had at least a moderate level of OCS (cutoff score 16), 7.56% had at least a severe level (cutoff score 24), and 1.31% had at least an extreme level (cutoff score 32).

**TABLE 1:** The clinical characteristics for the whole sample and different groups.

Groups	*n*	Male/female	Total CY-BOCS (mean ± SD)	Obsession (mean ± SD)	Compulsion (mean ± SD)
The whole sample	367	293/74	3.93 ± 5.15	1.30 ± 2.90	2.68 ± 3.73
TS group	152 (41.42%)	126/26	4.32 ± 5.32	1.53 ± 2.96	2.85 ± 3.83
Younger adolescents	230 (62.67%)	183/47	3.31 ± 4.53	1.04 ± 2.53	2.34 ± 3.51
Older adolescents	137 (37.33%)	110/27	6.02 ± 6.49	2.18 ± 3.81	3.84 ± 4.22

*CY-BOCS, Children’s Yale–Brown Obsessive–Compulsive Scale; SD, standard deviation; TS, Tourette syndrome; younger adolescents, aged <10 years; older adolescents, aged ≥10 years.*

### The Incident Rate of Obsessive–Compulsive Symptom in Tic Disorders

For the whole sample, the incident rate of OCS in TDs was 24.80%. The criteria of the OCS are a CY-BOCS score greater than 7 (cutoff of subclinical level). First, we calculated the incidence rate of OCS in different types of tic disorders [provisional tic disorders (PTDs), chronic motor or vocal tic disorder, and TS]. We also compared the differences among these different types (*p* < 0.01). The TS group showed a higher incidence rate of OCS in TDs (33.55%), followed by the chronic motor or vocal TD group (28.00%) and the provisional TD group (10.43%). Second, we compared the incidence rate in different gender groups and age groups. Men with TDs showed a higher incidence rate of OCS (*p* = 0.03), and the younger adolescent group showed a higher incidence rate of OCS (*p* < 0.01). For more details, see Table 2.

**TABLE 2:** The incident rate of OCS in TDs.

Different groups		Number of TD with OCS/total number	Subclinical OCS rate (%)	χ^2^	*p*-value
Different types of tic disorders	Provisional tic disorder	12/115	10.43	19.52	*p* < 0.01**
	Chronic motor or vocal tic disorder	28/100	28.00		
	Tourette syndrome	51/152	33.55		
Different age groups	Younger adolescents group	69/230	30.00	8.95	*p* < 0.01**
	Older adolescents group	22/137	16.06		
Different gender groups	Males group	80/293	27.30	4.90	*p* = 0.03*
	Females group	11/74	14.86		

Younger, aged <10 years; older, aged ≥10 years; TDs, tic disorders; OCS, obsessive–compulsive symptoms. *p < 0.05; **p < 0.01.

### Associations Between Obsessive–Compulsive Symptom and Tic Symptoms

We evaluated the discriminant validation of the CY-BOCS based on the Pearson correlations of the total tic severity with the obsession, compulsion, and total CY-BOCS scores. The results showed no significant correlations (*p* > 0.05). In addition, the Pearson correlation between YGTSS impairment and obsession, compulsion, and total CY-BOCS scores showed significant results (*p* < 0.05; Table 3).

**TABLE 3:** The correlation analysis between the CY-BOCS based on YGTSS.

CY-BOCS and subscales	YGTSS		
	Total tic severity	Impairment	Total YGTSS score
Obsession	0.08	0.19*	0.18*
Compulsion	0.11	0.11	0.14
CY-BOCS total	0.11	0.21*	0.21*

*p < 0.05.

### Item Correlations With the Total Children’s Yale–Brown Obsessive–Compulsive Scale Score

We examined the correlation between each item score and the total CY-BOCS score in patients with TDs. All item, obsession, and compulsion scores were significantly correlated with the total CY-BOCS score (*p* < 0.001). Regarding the correlation between the total score of the CY-BOCS and individual items, O3, C2, and C3 showed lower correlations (Supplementary Table 2).

### Reliability of the Children’s Yale–Brown Obsessive–Compulsive Scale

The Cronbach’s alpha values for the total sample, TS group, younger adolescent group, and older adolescent group were 0.81, 0.81, 0.77, and 0.86, respectively. A total of 30 participants who were randomly selected (random number method) from the whole sample were recalled to complete the 1-month retest. The test-retest reliability of the CY-BOCS was 0.82 (*p* = 0.02).

### Confirmatory Factor Analysis of Children’s Yale–Brown Obsessive–Compulsive Scale in Different Groups

**FIGURE 1:**
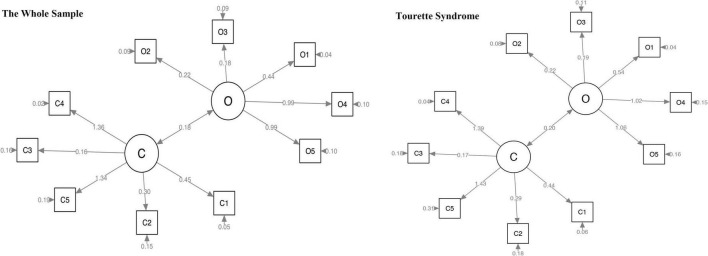
Confirmatory factor analysis of CY-BOCS in the whole sample and the TS group. CY-BOCS, Children’s Yale–Brown Obsessive–Compulsive Scale; TS, Tourette syndrome.

**FIGURE 2:**
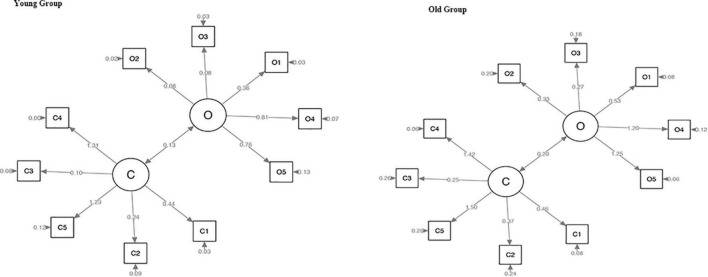
Confirmatory factor analysis of CY-BOCS in the younger and older adolescent groups. CY-BOCS, Children’s Yale–Brown Obsessive–Compulsive Scale.

We calculated the construct validity of the CY-BOCS by CFA in the whole sample and the TS group, with comparative fit index (CFI) values of 0.84 and 0.85, respectively. The CFI values in the younger and older adolescent groups were 0.94 and 0.74, respectively (Table 3 and Figures 1, 2). The criteria of the model fit index are included in Table 4.

**TABLE 4:** The results of CFA for different groups.

Model	Variable	The whole sample (*n* = 367)	TS group (*n* = 152)	Young group (*n* = 230)	Old group (*n* = 137)	The model fit criteria
	χ^2^/df	7.65	4.9	4.72	9.47	<5, good; <8, accept level
Two-factor model	CFI	0.84	0.86	0.94	0.74	>0.9, good; 0.8–0.9, accept level
	TLI	0.80	0.81	0.92	0.66	>0.9, good; 0.8–0.9, accept level
	AIC	4218.87	1931.38	1315.97	2191.48	N/A
	BIC	4300.88	1993.74	1388.17	2252.80	N/A
	RMSEA	0.09	0.20	0.08	0.29	<0.1
	SRMR	0.04	0.09	0.07	0.14	<0.1

*CFI, comparative fit index; TLI, Tucker–Lewis index; AIC, Akaike; BIC, sample-size adjusted Bayesian; RMSEA, root mean square error of approximation; SRMR, standardized root mean square residual.*

## Discussion

This is the first study to investigate the OCS of TDs in China based on a large sample. We found that the mean OCS in TDs was subclinical (the criterion of subclinical OCS: the total score of CY-BOCS was less than 8). However, more OCSs were identified in the TS group and older adolescent group with TDs. It should be noted that OCS is an important indicator of the function of patients with TDs. The age might be one of the influential factors for the OCS in TDs. Furthermore, the CY-BOCS showed good psychometric properties in patients with TDs. The CFA results showed that the two-factor structure of CY-BOCS was “stable” in younger patients (less than 10 years).

Children’s Yale–Brown Obsessive–Compulsive Scale has been widely used for the assessment of OCS in TDs. Previous studies have demonstrated the fine psychometric properties of CY-BOCS in the assessment of patients with OCD (22, 23). For instance, the internal consistency was high and had a good inter-rater agreement for the subscale and total OCD (29). However, no studies have investigated the psychometric properties of CY-BOCS in patients with TDs. Take Cronbach’s alpha as an example, we found that the Cronbach’s alpha for the older adolescent group with TDs was 0.86 in the present study, which was at the same level as other versions in patients with OCD (the internal consistency of children with OCD was 0.86 for the Korean version and 0.87 for the Spanish version) (30, 31). Based on these results, we provided evidence for the reliability and validity of the CY-BOCS, which will benefit future studies that assess the OCS in TS.

Indeed, tic symptoms and OCS are closely related and overlap in some respects, such as epidemiology, anatomy, genetics, and treatment (32). Due to the close association between OCS and TDs, assessing OCS in patients with TDs is an important issue in future studies (13). In the present study, we found an association between OCS and functional impairment in patients with TDs. As one of the comorbidities of TDs, OCS may be regarded as one of the outcomes of TD (11). Although the CY-BOCS is not a diagnostic instrument for childhood OCD, it is helpful in assessing the severity of OCS and evaluating the treatment response. The CY-BOCS rates on specific items of obsession and compulsion will help the clinicians to better understand the symptoms of pediatric patients with TD comorbid OCD. Therefore, following the European guidelines for TS-related disorders, CY-BOCS can be recommended as an assessment tool for TDs in China.

In the present study, items O3 and C3 showed weak associations with the total CY-BOCS. A possible reason might lie in the cultural differences between the East and the West. For example, compared with Westerners, Chinese people prefer to conceal their feelings and express fewer inner feelings (33). Moreover, this study was performed in patients with TDs rather than OCD. This indicates that TDs may show different features of OCS in different cultures. We can consider performing some comparisons of OCS in TDs across different cultures in the future.

In the present study, OCS severity was significantly higher in the older group than in the younger group. There were three reasons for the higher OCS in the older group. First, this difference was probably due to the longer TD duration in the older group when compared with that in the younger group. Second, older patients may report OCS more clearly. Third, there was an age effect of the OCS in TDs. For example, when we controlled for the potential influence of the duration of illness, the severity of OCS still showed an association with age. This result indicated that there may be an “age effect” for OCS in TDs. For the TS groups that showed more OCS, the most likely reason was that with the persistence or the higher severity of tic symptoms, more OCS was developed. Higher levels of cognitive and metacognitive beliefs have been reported in adolescents with OCD than in children with OCD (34). Notably, OCS often appears 2 years after the onset of tic symptoms (35). The results of the present study also demonstrated that the CY-BOCS is a reliable and validated scale for assessing symptom severity in young patients with TDs (age 6–9 years) (CFI = 0.94).

Overall, age might have an important influence on OCS severity in patients with TDs. A higher level of severity with a lower incidence rate of OCS was identified in older adolescents with TDs. The clinical implications of these results show that the OCS should be given more attention to men with TDs. Moreover, in different types of TDs, the TS might have more OCS. Studies with larger sample sizes are warranted to confirm the effect of age and gender on OCS in TDs. For the assessment of OCS in TDs, CY-BOCS might be an important tool with good psychometric properties that can be widely used in TDs.

This study had several limitations, which should be addressed in future studies. First, the main limitation of the present study is that it has no concurrent validity measures for the CY-BOCS. Due to the lack of another tool for the assessment of OCS, we could not calculate the concurrent validity of the CY-BOCS. Other scales may be better suited for the assessment of obsession and compulsion. Second, the sample only included children and not the entire age range, such as adults. Third, the sample size was small. Therefore, additional studies with larger sample sizes are needed to confirm these results. Fourth, we only focused on the OCS in TDs, while other comorbidities of TDs, such as ADHD, SIB, and anxiety, were not considered in this study. More studies are needed in the future to research the other comorbidities of TDs. In addition, we did not consider the impaired function by OCS in patients with TDs. In future studies, it would be interesting to evaluate the quality of life, cognitive profile, executive functions, and the emotional–behavioral profile of TD patients with OCS. We can also compare the differences between the TDs with and without OCS in these dimensions.

## Conclusion

More OCSs were identified in the TS group and older adolescent group with TDs. The CY-BOCS showed good psychometric properties in children and adolescents with TD, especially in younger patients with TD. OCS in TDs might be influenced by age and gender and different types of TDs. Further studies are needed to explore the OCS in TDs, especially for TS.

## Data Availability Statement

The raw data supporting the conclusions of this article will be made available by the authors, without undue reservation.

## Ethics Statement

The studies involving human participants were reviewed and approved by the Ethics Committees of Beijing Children’s Hospital of Capital Medical University. Written informed consent was obtained from the individual(s), and minor(s)’ legal guardian/next of kin, for the publication of any potentially identifiable images or data included in this article.

## Author Contributions

YC and YL took the initiative. YG and MW finished the data collection. YL performed the data analysis. JY finished the draft. All authors have read and approved the manuscript.

## Conflict of Interest

The authors declare that the research was conducted in the absence of any commercial or financial relationships that could be construed as a potential conflict of interest.

## Publisher’s Note

All claims expressed in this article are solely those of the authors and do not necessarily represent those of their affiliated organizations, or those of the publisher, the editors and the reviewers. Any product that may be evaluated in this article, or claim that may be made by its manufacturer, is not guaranteed or endorsed by the publisher.
