# Supplementary material for: The Obsessive–Compulsive Symptoms in Tic Disorders and the Psychometric Properties of Children’s Yale–Brown Obsessive–Compulsive Scale: An Evidence-Based Survey in a Chinese Sample
Source: Front Pediatr. 2022 Jun 9;10:794188. doi: 10.3389/fped.2022.794188 (PMC9218257; doi:10.3389/fped.2022.794188)
Supplement: Supplementary file 1 [file Table_1.docx]

**Table S1. The Shapiro-Wilk test for CY-BOCS**

| **Items** | **Statistic** | **df** | ***p* value** |
| --- | --- | --- | --- |
| **C 1** | **0.64** | **367** | **< 0.001** |
| **C 2** | **0.51** | **367** | **< 0.001** |
| **C 3** | **0.32** | **367** | **< 0.001** |
| **C 4** | **0.70** | **367** | **< 0.001** |
| **C 5** | **0.69** | **367** | **< 0.001** |
| **Compulsion** | **0.72** | **367** | **< 0.001** |
| **O 1** | **0.50** | **367** | **< 0.001** |
| **O 2** | **0.27** | **367** | **< 0.001** |
| **O 3** | **0.25** | **367** | **< 0.001** |
| **O 4** | **0.49** | **367** | **< 0.001** |
| **O 5** | **0.48** | **367** | **< 0.001** |
| **Obsession** | **0.51** | **367** | **< 0.001** |
| **Total Scores** | **0.77** | **367** | **< 0.001** |

***Note: CY-BOCS, Children’s Yale-Brown Obsessive-Compulsive Scale. C1-C5, O1-O5: the ten items of CY-BOCS.***
