# Supplementary material for: The Obsessive–Compulsive Symptoms in Tic Disorders and the Psychometric Properties of Children’s Yale–Brown Obsessive–Compulsive Scale: An Evidence-Based Survey in a Chinese Sample
Source: Front Pediatr. 2022 Jun 9;10:794188. doi: 10.3389/fped.2022.794188 (PMC9218257; doi:10.3389/fped.2022.794188)
Supplement: Supplementary file 2 [file Table_2.docx]

**Table S2. The Items Correlation with Total CY-BOCS (Spearman Rank Correlation)**

| **Items** | **Mean ± SD** | **Correlation with**  **CY-BOCS Total** |
| --- | --- | --- |
| **O 1** | **0.41 ± 0.51** | **0.62**** |
| **O 2** | **0.24 ± 0.51** | **0.39**** |
| **O 3** | **0.13 ± 0.6** | **0.35**** |
| **O 4** | **1.05 ± 1.38** | **0.61**** |
| **O 5** | **1.03 ± 1.51** | **0.62**** |
| **Obsession** | **2.85 ± 3.82** | **0.62**** |
| **C 1** | **0.28 ± 0.53** | **0.82**** |
| **C 2** | **0.09 ± 0.31** | **0.63**** |
| **C 3** | **0.09 ± 0.37** | **0.42**** |
| **C 4** | **0.54 ± 1.05** | **0.84**** |
| **C 5** | **0.53 ± 1.08** | **0.84**** |
| **Compulsion** | **1.53 ± 2.96** | **0.85**** |

*Note: SD, Standard Deviation; CY-BOCS, Children’s Yale-Brown Obsessive-Compulsive Scale; ^**^, p <0.001; O1: Time Occupied by Obsessive Thoughts; O2: Interference Due to Obsessive Thoughts; O3: Distress Associated with Obsessive Thoughts; O4: Resistance Against Obsessions; O5: Degree of Control Over Obsessive Thoughts; C1: Time Spent Performing Compulsive Behaviors; C2: Interference Due to Compulsive Behaviors; C3: Distress Associated with Compulsive Behaviors; C4: Resistance Against Compulsions; C5: Degree of Control Over Compulsive Thoughts.*
